# Elevated microRNA-21 Is a Brake of Inflammation Involved in the Development of Nasal Polyps

**DOI:** 10.3389/fimmu.2021.530488

**Published:** 2021-04-15

**Authors:** Ruowu Liu, Jintao Du, Jiao Zhou, Bing Zhong, Luo Ba, Jie Zhang, Yafeng Liu, Shixi Liu

**Affiliations:** ^1^ Department of Otolaryngology-Head and Neck Surgery, West China Hospital, Sichuan University, Chengdu, China; ^2^ Upper Respiratory Tract Laboratory of Department of Otolaryngology-Head and Neck Surgery, West China Hospital, Sichuan University, Chengdu, China; ^3^ State Key Laboratory of Biotherapy, West China Hospital, Sichuan University, Chengdu, China; ^4^ Department of Otolaryngology, People’s Hospital of the Tibet Autonomous Region, Lasha, China

**Keywords:** cytokines, nasal epithelial cells, PDCD4, miR-21, chronic rhinosinusitis, nasal polyps

## Abstract

**Background:**

CRSwNP is an inflammatory disease but the mechanism is not yet fully understood. MiR-21, a member of miRNAs, has been reported to play roles in mediating inflammation. However, the expression of miR-21 and its role in patients with CRSwNP remain elusive.

**Methods:**

Turbinates from control subjects, uncinate processes from CRSsNP, polyp tissues from CRSwNP, and nasal epithelial cells brushed from nasal mucosa were collected. The expression of miR-21 and cytokines in nasal tissues and epithelial cells were detected by qPCR. The localization of miR-21 was detected by ISH, and its target was identified by bioinformation analysis, qPCR, IHC, WB, and luciferase reporter system. The protein and mRNA of PDCD4 and NF-κB P65 were determined by WB and qPCR after miR-21 transfection in HNEpC. The role of miR-21 on cytokines was analyzed in HNEpC and nasal polyp explants.

**Results:**

MiR-21 was upregulated in CRSwNP relative to control subjects by qPCR, which was determined mainly in nasal epithelial cells of CRSwNP by ISH. Both pro-inflammation cytokines (IL-1β, IL-6, IL-8, IL-25, and TSLP) and a suppressive cytokine (IL-10) were overexpressed in the epithelial cells of CRSwNP. The expression of miR-21 was positively correlated with IL-10 and negatively correlated with IL-6, IL-8, IL-33, and TSLP in the epithelial cells of CRSwNP. As a potential target of miR-21, the expression of PDCD4 was negatively correlated with miR-21 in CRSwNP. In HNEpC, miR-21 could reduce the expression of PDCD4 at both mRNA and protein levels, and bioinformation analysis and luciferase reporter system confirmed PDCD4 as one target of miR-21. Furthermore, miR-21 could decrease the activation of NF-κB and increase IL-10 mRNA. Both SEB and LPS could elevate miR-21, with IL-25, IL-33, TSLP induced by SEB and IL-1β, IL-6, IL-8 induced by LPS, while the miR-21 could regulate the expression of IL-33, TSLP, IL-1β, IL- 6 and IL-8 *in vitro* and *ex vivo*. Clinically, miR-21 expression was inversely correlated with the Lund-Mackay CT scores and the Lund-Kennedy scores in CRSwNP.

**Conclusion:**

MiR-21 could be a prominent negative feedback factor in the inflammation process to attenuate the expression of pro-inflammatory cytokines, thereby playing an anti-inflammation role in CRSwNP.

## Introduction

Chronic rhinosinusitis (CRS) is a common inflammatory disease with approximately 10% of the adult population affected all over the world (1, 2). CRS is divided into two types based on the presence or absence of nasal polyp (NP): CRS without NP (CRSsNP) and CRS with NP (CRSwNP) (3). CRSwNP is a heterogeneous disease involved in a variety of structural cells, immune cells, and inflammatory mediators (4, 5). The etiology and pathophysiology of NP are intricate and nasal epithelial cells are known to play an important role (3). The nasal epithelium is exposed to a variety of stimuli like SEB and LPS, which cause dysfunction of the epithelium barrier and activation of epithelial cells. Cytokines, such as IL-1β, IL- 6, IL-8, IL-25, IL-33, and TSLP are secreted by activated epithelial cells and can affect numerous immune cells, inciting inflammatory responses (6–8). However, inflammation of CRSwNP is not unlimitedly aggravating, and the functions of cytokines are strictly controlled to prevent their overaction through several mechanisms (9). IL-10 is an anti-inflammatory cytokine that can limit and ultimately terminate inflammatory responses and could be expressed by nasal epithelial cells (10, 11). Although the anti-inflammatory regulation of NP has been studied in recent years, the complicated mechanism remains elusive.

MicroRNAs (miR) are a class of small noncoding RNAs that regulate the expression of target genes by preventing the translation of target mRNAs or inducing their degradation, or both, depending on the level of complementarity (12). MicroRNAs are involved in the regulation of immune systems and inflammation (13, 14), and increasing studies show that miRNAs play an important role in the pathogenesis of NP (15–18). MiR-21, a member of microRNAs, is highlighted as potentially playing a pivotal role in regulating inflammation (19–21). Notably, miR-21 may play a dynamic role in inflammatory responses, both pro-inflammatory and dampen inflammation (21). Recent studies have found that miR-21 was upregulated in asthmatic children and multiple models of experimental asthma and downregulated in cord blood monocytes from allergic rhinitis children (22–24), which indicates miR-21 is associated with airway inflammatory diseases. However, the expression and role of miR-21 in patients with CRSwNP are unclear.

In this study, we identified the expression and location of miR-21 and the correlations between miR-21 and cytokines in CRSwNP. Next, we validated the expression of PDCD4, a target gene of miR-21, in CRSwNP. Furthermore, we examined the role of miR-21 on the nasal epithelial cells and nasal polyp explants. Finally, we evaluated the relationship between miR-21 and disease severity.

## Methods

### Subjects and Sample Tissues

Subjects were recruited into the study based on the diagnostic criteria of CRS including medical history, physical examination, nasal endoscopy, and computed tomography (CT) scan of the sinuses (3). Unilateral rhinosinusitis, antrochoanal polyps, allergic fungal rhinosinusitis, cystic fibrosis, or immotile ciliary disease were excluded from the study. Before surgery, the severity of nasal symptoms was evaluated according to SNOT-20, Lund-Mackay CT scores, and Lund-Kennedy endoscopic scores (3). The atopic status of the patient was confirmed by a skin prick test. The diagnosis of asthma was performed by the pneumologist. Patients had not used oral or nasal corticosteroids and topical medication for more than 4 weeks before surgery. Turbinate tissues were obtained from control subjects during septoplasty. Uncinate process tissues and NP tissues were collected from patients with CRSsNP and CRSwNP respectively through functional endoscopic sinus surgery. All samples were divided into 3 parts: one part was preserved in RNA-latter and snap-frozen at -20°C for total RNA isolation; and the other part was fixed in 4% paraformaldehyde, gradiently dehydrated, and subsequently embedded in paraffin wax or frozen in O.C.T. Compound (SAKURA, Northbrook, USA) at -80°C. The remainder was immediately frozen in liquid nitrogen for isolation of proteins.

Patient characteristics are shown in **Table 1**. This study was approved by the Medical Ethics Committee of the West China Hospital of Sichuan University (No.: WCH2015-199) and was conducted in accordance with approved institutional guidelines. Informed written consent was obtained from all patients prior to the study.

**Table 1:** Patient characteristics and symptom scores.

	Control	CRSsNP	CRSwNP
N	15	10	45
Age, median (range)	33(18-46)	43(19-57)	46(18-62)
Female/male	1/14	3/7	19/26
Asthma (n)	0	0	6
SPT* (n)	0	3	14
SNOT-20, median (range)	2(0-20)	11.5(2-36)	9.5(1-29)
L-K^#^, median (range)	0	5.5(3-10)	9(2-12)
L-M^$^, median (range)	0	6(0-20)	15(4-24)

*skin prick test; ^#^Lund-Kennedy score; ^$^Lund-Mackay CT score.

### Isolation of Nasal Epithelial Cells From Nasal Tissues

Nasal epithelial cells (NECs) were obtained by brushing the surface with turbinate of 10 control subjects and NP of 17 CRSwNP respectively before functional endoscopic sinus surgery. The cells were collected from each subject’s nostrils using nasal brushing.

### Isolation of Total RNA and Real-Time Quantitative PCR

The total RNA for the specimens was isolated using miRCURY™ RNA Isolation Kit–Tissue (Exiqon, Vedbaek, Denmark), and the total RNA from cells was extracted using RNAiso Plus (Takara Biotechnology, Dalian, China) according to the manufacturer’s instructions. MiRNAs were reverse transcribed to cDNA using All-in-One™ miRNA First-Strand cDNA Synthesis Kit (GeneCopoeia, Guangzhou, China). The real-time quantitative PCR (qPCR) of miRNAs was performed with All-in-One™ miRNA qRT-PCR Detection Kit (GeneCopoeia). We synthesized mRNAs to cDNA using Bimake™ All-in-One cDNA Synthesis SuperMix (Bimake, Houston, USA), and the qPCR of mRNAs was performed with Bimake™ 2x SYBR Green qPCR master mix (Bimake). Relative expression was calculated with the 2^−ΔΔCT^ method and levels were normalized using U6 and GAPDH for miRNAs and mRNAs, respectively. The primer sequences used in this study are shown in **Table 2**.

**Table 2:** The primer sequences used in qPCR.

Primer	Forward (5’-3’)	Reverse (5’-3’)
GAPDH	CATCAAGAAGGTGGTGAATC	TCAAAGGTGGAGGAGTGGGC
IL-1β	AGCTACGAATCTCCGACCAG	CGTTATCCCATGTGTCGAAGA
IL-6	CAACCTGAACCTTCCAAAGATG	ACCTCAAACTCCAAAAGACCAG
IL-8	AAGGTGCAGTTTTGCCAAGG	CAACCCTCTGCACCCAGTTT
IL-10	GCCTAACATGCTTCGAGAT	AGTCTATAGAGTCGGCACC
IL-25	GGCTGTACCGTGTTTCCTAAG	CTTCATGGCAAGTGGTTGTAG
IL-33	AGGAGAGAAACCACCAAAG	CTGGACCCTGATATACCAAAG
TSLP	ATGTTCGCCATGAACTAAGGC	GCGACGCCACAATCCTTGTA
PDCD4	TGGATGTCCCACATTCATACTCT	TCTGGTTAAGACGACCTCCATCT
NF-κB	ATGTGGAGATCATTGAGCAGC	CCTGGTCCTGTGTAGCCATT

### Fluorescence *In Situ* Hybridization

The frozen specimens of the O.C.T. Compound were made into 8µm-thick cryosections. Then, *in situ* hybridization (ISH) was performed using the Enhanced Sensitive ISH Detection kit V(FITC) (Boster, Wuhan, China) according to the protocol recommended by the manufacturer. Locked nucleic acid (LNA) probes with 5`-DIG and 3`-DIG double labeled were used for ISH. The detection probe of miR-21 was purchased from Exiqon. Positive control U6 and negative scrambled LNA probes were synthesized by Takara.

### Histological Analysis

The specimens were embedded in paraffin wax then prepared as 4µm-thick sections. The sections of CRSwNP were stained with hematoxylin and eosin to determine eosinophil infiltration. Three random high power fields were selected, and the number of eosinophils and total inflammatory cells were counted. CRSwNP can be classified into two groups: eosinophilic CRSwNP (E-CRSwNP), which was defined as the percentage of tissue eosinophils that exceeded 10% of total infiltrating cells, and non-eosinophilic CRSwNP (NE-CRSwNP), which did not fulfill this criterion (25). Sections were air-fixed onto microscope glass slides and air-dried overnight at 37°C, followed by deparaffinization, hydration. Sections were then treated with 3% hydrogen peroxide at room temperature for 10min and incubated with rabbit polyclonal antibody against PDCD4 (Proteintech, Wuhan, China) at 4°C overnight. Thereafter, sections were incubated with biotinylated secondary antibody at 37°C for 30min. Immunohistochemistry (IHC) staining was performed using Biotin-Streptavidin horseradish peroxidase Detection Kit (SP-9001, ZSGB-Bio, Beijing, China) and DAB Detection Kit (ZLI-9061, ZSGB-Bio). Three fields of view were selected for each section and images were collected under a 400-fold microscope. The mean optical density (MOD) of each image was measured through Image-Pro Plus 6.0 image analysis system, and the average value of three images of one sample is the MOD of each section.

### Western Blotting

After being thoroughly grounded in liquid nitrogen, the tissues were collected for western blotting (WB). The total protein of tissues or cells was extracted in RIPA lysis buffer with a cocktail of protease inhibitors (Bimake), and then boiled for 10 min with SDS loading buffer. Equal amounts of protein were electrophoresed on SDS-PAGE in 10% Tris‐glycine gels and transferred to PVDF membranes (Millipore, MA, USA). Membranes were blocked with 5% non-fat milk in tris buffered solution containing 0.05% Tween-20 at room temperature for 1 h, followed by overnight incubation with primary antibodies against β-actin (Beyotime, Beijing, China), PDCD4 (Proteintech, Wuhan, China), phosphorylated NF-κB P65 (p-P65) and NF-κB P65 (P65) (Cell Signaling Technology, Danvers, USA). After washing thrice at room temperature, the membranes were incubated with secondary antibody (Zen Bioscience, Chengdu, China) and signals were visualized by using ECL Plus Western Blotting Reagent Pack (Bio-Red, Hercules, USA). The band intensities were quantified by Fusion Solo Imaging System (VIBER LOURMAT, FRANCE).

### Nasal Epithelial Cell Line Culture and Transfections

The human nasal epithelial cell (HNEpC) line used in this experiment was gifted to our laboratory by the first affiliated hospital of Sun Yat-sen University. The HNEpC cells were maintained in RPMI-1640 medium containing 10% fetal bovine serum, 100 U/mL penicillin, and 100 ug/mL streptomycin (Gibco, Paisley, UK), and incubated at 37°C with 5% CO2. When a 70–80% confluent monolayer appeared, the cells were used for experiments. To investigate the role of miR-21 in HNEPC, transfection was performed. MiR-21 mimics, inhibitors, and negative controls were synthesized by Genepharma (GenePharma, Shanghai, China). 50nM miR-21 mimic (Mimics), 50nM mimic negative control (mNC), 50nM miR-21 inhibitor (Inhibitor), and 50nM inhibitor negative control (iNC) was transfected into the cells using EndoFectin™ Max (GeneCopoeia, Guangzhou, China) for 24h, according to the manufacturer’s instruction. Then, cells were treated with SEB (Toxin Technology, Sarasota, FL, USA) at concentrations (125/250/500 ng/ml) and LPS (Biosharp, Hefei, China) at concentrations (10/100/1000 ng/ml) for 24h. Meanwhile, cells were stimulated at various times with 250 ng/ml of SEB and 1 ug/ml of LPS. Furthermore, cells were treated with either 250ng/ml SEB or 1ug/ml LPS after miR-21 transfection. All cell groups were harvested for qPCR analysis or WB. Experiments were repeated three times.

### Isolated Nasal Polyp Explant Culture and Transfections

Nasal polyp tissues were isolated and cultured as described previously (26). In brief, fresh nasal polyp tissues from six CRSwNP patients were washed and suspended in RPMI-1640 medium containing 50 IU/ml penicillin, 50 mg/mL streptomycin, and 0.1% bovine serum albumin. Then the tissue was cut into smaller pieces and passed through a mesh (pore size 0.9 mm^2^) to obtain tissue fragments. Following three washings, the tissue fragments were resuspended as 0.04 g tissue/ml culture medium, and then the fragments were divided into a 24-well plate for explant culture. MiR-21 agomir (upgrade miR-21 mimics) and antagomir (upgrade miR-21 inhibitor) were synthesized by Genepharma. 50nM miR-21 agomir and 50nM miR-21 antagomir were transfected into explants using EndoFectin™ Max, according to the manufacturer’s instruction. Then, explants were treated with either 250ng/ml SEB or 1ug/ml LPS after transfection for 24h. Explants were collected from each well for qPCR analysis.

### Bioinformatics Prediction and Luciferase Reporter System

The target genes of miR-21 were predicted by the following tools: TargetScan (http://www.targetscan.org/), miRanda (http://www.microrna.org/) and PicTar (http://pictar.mdc-berlin.de/). The luciferase reporter gene pGL3-eGFP-WT PDCD4 3′-UTR was generated, which contains the 3′-UTR of wild-type PDCD4, and was predicted to contain a potential miR-21 binding site (microRNA recognition element, MRE). A mutant PDCD4-1 3′-UTR at the MRE was also constructed (pGL3-eGFP-mutant PDCD4-1 3′UTR). The pRL-TK vector was used as an internal control reporter and used in combination with the experimental reporter vector to co-transfect cells.

### Statistical Analysis

Statistical analyses were performed by using GraphPad Prism 8.0 (GraphPad Software, San Diego, USA). Data were presented as mean ± standard error of mean. For nasal tissues, unpaired comparisons between multiple groups were tested by using the Kruskal–Wallis test, and the unpaired comparisons between the two groups were calculated by Mann–Whitney *U* test. One-way ANOVA was carried out to compare the differences in cell culture data. The Spearman correlation coefficient was used to determine variable relationships. Asterisk indicates statistical significance (*P<0.05, **P<0.01, ***P<0.001).

## Results

### The Expression and Location of miR-21 in Nasal Mucosa and NP

**Figure 1:**
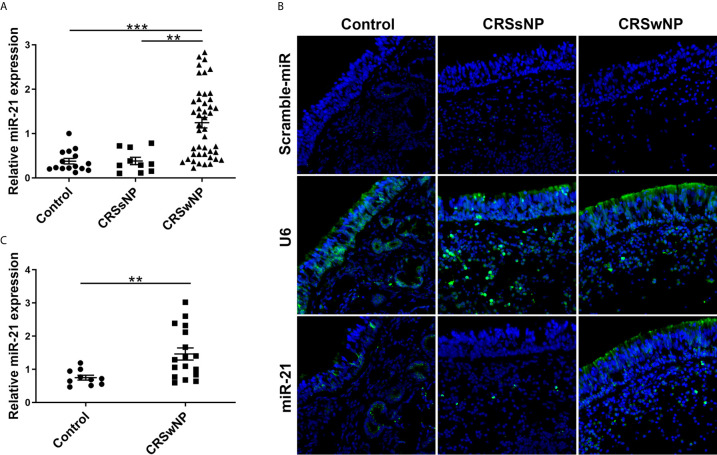
The expression and location of MiR-21 in different nasal tissues. **(A)** The expression of miR-21 was analyzed by qPCR assay among control subjects (n=15), CRSsNP (n=10) and CRSwNP (n=45). **(B)** The distribution pattern of miR-21 was investigated by fluorescence *ISH* in the control group, CRSsNP, and CRSwNP. Scramble-miR means negative control, U6 means positive control. **(C)** The expression of miR-21 in NECs was analyzed by qPCR assay between control subjects (n=10) and CRSwNP (n=17). Kruskal–Wallis test and Mann–Whitney *U* test was used for comparison among multiple groups and between two groups. The asterisk indicates statistical significance, **P < 0.01, ***P < 0.001.

To determine the expression level of miR-21, we performed a qPCR assay among 15 control subjects, 10 patients with CRSsNP, and 45 patients with CRSwNP according to the method described previously (27). The qPCR analysis showed miR-21 was significantly increased in CRSwNP compared with control subjects and CRSsNP (P<0.001), but it was not different between control subjects and CRSsNP (P>0.05) (**Figure 1A**). After being classified into two groups, the qPCR results showed miR-21 was significantly increased in E-CRSwNP and NE-CRSwNP compared with control subjects, but it was not different between E-CRSwNP and NE-CRSwNP (**Supplementary Figure 1**). We next sought to investigate the distribution pattern of miR-21 in nasal mucosa by fluorescence *in situ* hybridization. According to the fluorescence images (**Figure 1B**), we found that miR-21 was mainly expressed in epithelial cells of both NP and normal mucosa, with stronger signals of miR-21 in CRSwNP than that in control subjects. In addition, miR-21 was localized to the submucosal immune cells of NP but the submucosal glands of normal mucosa. The expression of miR-21 in CRSsNP was barely detected except for a few weak signals in the submucosa. Then, we detected the expression level of miR-21 in NECs from 10 control subjects and 17 patients with CRSwNP. The qPCR analysis showed that miR-21 was increased in the NECs of CRSwNP compared with that of the control subjects (P<0.01) (**Figure 1C**).

### Expression of Epithelial Cytokines in Nasal Tissues and Nasal Epithelial Cells

**Figure 2:**
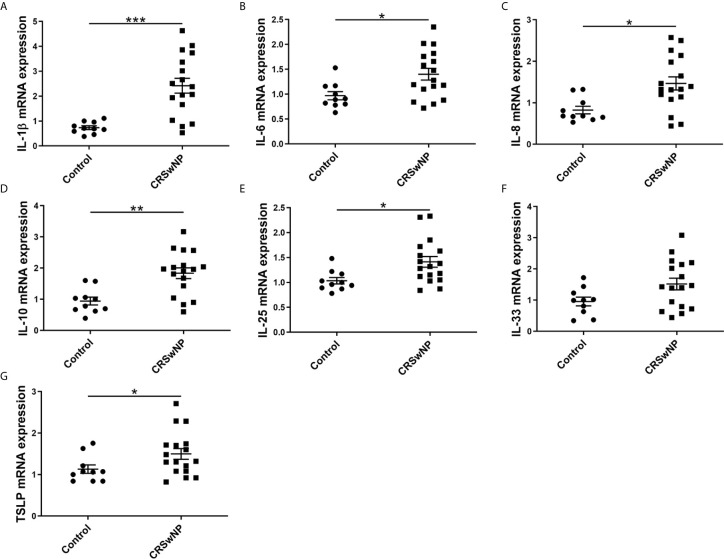
The relative expression level of epithelial cytokines in the epithelial cells of NPs. The mRNA expression of **(A)** IL-1β, **(B)** IL-6, **(C)** IL-8, **(D)** IL-10, **(E)** IL-25, **(F)** IL-33, and **(G)** TSLP in NECs were measured by qPCR and compared between control subjects (n=10) and CRSwNP (n=17). Mann–Whitney U test was used for comparison between the two groups. The asterisk indicates statistical significance, *P < 0.05, ** P < 0.01, ***P < 0.001.

The mRNA expressions of IL-1β, IL-6, IL-8, IL-10, IL-25, IL-33, and TSLP among control subjects, CRSsNP and CRSwNP were assessed using qPCR. The expression levels of IL-1β, IL-6, IL-10, and IL-25 in CRSwNP were significantly higher than in control subjects (P<0.05). However, no significant difference in IL-8, IL-33, and TSLP was found between control subjects and CRSwNP (P>0.05) (**Supplementary Figure 2**). Next, the expression of these cytokines in NECs from control subjects and CRSwNP were examined. IL-1β, IL-6, IL-8, IL-10, IL-25, and the TSLP of NECs were significantly higher expressed in CRSwNP than in control subjects (P<0.05), and no significant difference of IL-33 was found in NECs between control subjects and CRSwNP (P>0.05) (**Figures 2A-G**).

### Correlations Between miR-21 Expression and Cytokines in Nasal Polyps and Epithelial Cells of NPs

**Figure 3:**
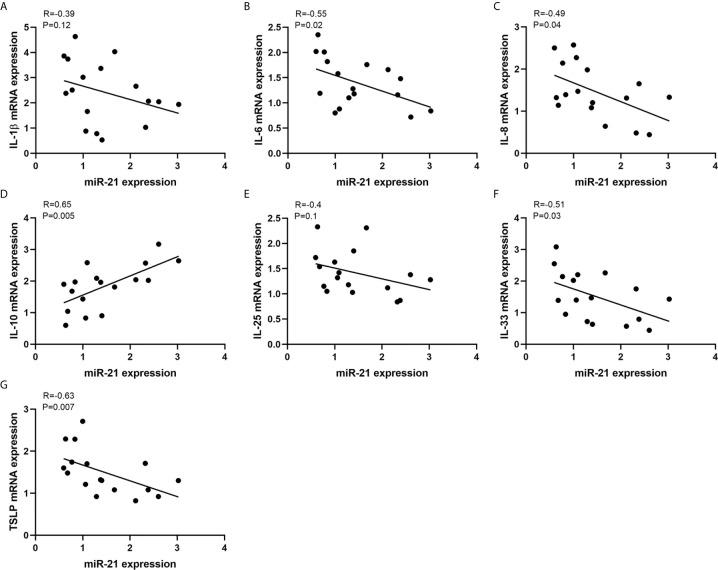
Correlations between miR-21 expression and cytokines. Correlations between miR-21 expression and mRNA levels of **(A)** IL-1β, **(B)** IL-6, ** (C)** IL-8, **(D)** IL-10, **(E)** IL-25, **(F)** IL-33, and **(G)** TSLP were investigated in NECs of NPs (n=17). R values indicate Spearman correlation coefficients.

To investigate the potent role of miR-21 in CRSwNP, the correlation between miR-21 expression and cytokines in CRSwNP was examined. Pearson correlation test showed that miR-21 may be positively related with IL-10 but be negatively related with IL-1β, IL-6, IL-8, IL-33, and TSLP in NP tissues, although the correlations were not significant (P>0.05) (**Supplementary Figure 3**). Then, the correlations between miR-21 expression and cytokines in E-CRSwNP and NE-CRSwNP were examined respectively, without significant differences in E-CRSwNP and NE-CRSwNP. (**Supplementary Figure 4**). Furthermore, the correlations between miR-21 expression and cytokines were examined in NECs of NP. The results showed that miR-21 was positively related with IL-10 and negatively related with IL-6, IL-8, IL-33, and TSLP (P<0.05). There was no significant correlation between miR-21 and IL-1β or IL-25 (P>0.05) (**Figure 3**). We thus speculated that upregulated miR-21 in the epithelium might have the function of anti-inflammation in CRSwNP.

### Expression of PDCD4 in Patients With CRSwNP

**Figure 4:**
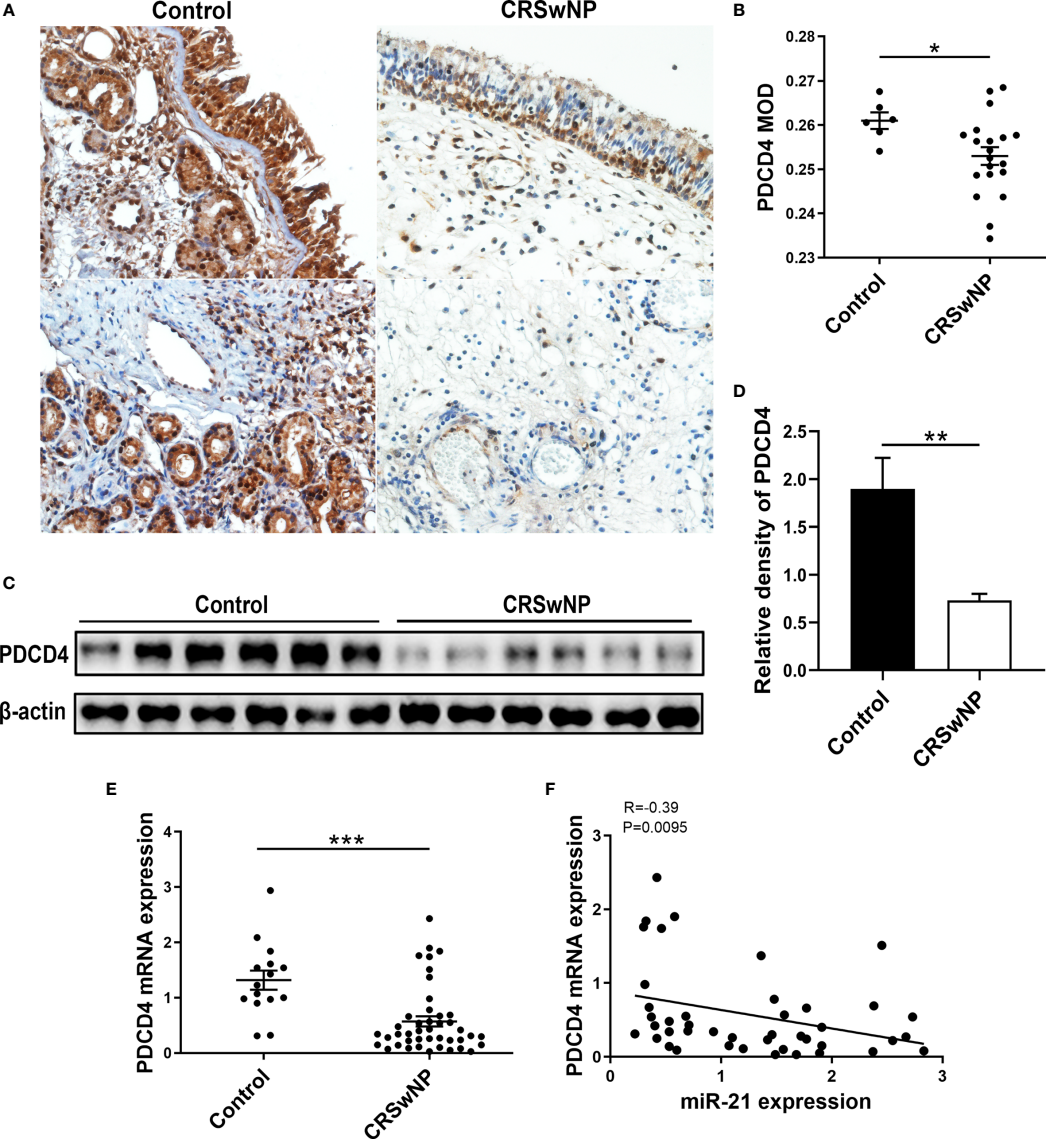
Expression of PDCD4 in patients with CRSwNP. **(A)** IHC staining of PDCD4 was performed in normal tissues and NPs. **(B)** Comparison of PDCD4 expression were assessed through mean optical density (MOD) between control subjects (n=6) and NPs (n=20). **(C)** PDCD4 protein expression was determined by WB in control subjects (n=6) and CRSwNP (n=6), with β-actin expression as a control. **(D)** Relative PDCD4 protein expression was quantified by densitometry based on immunoblot images. **(E)** Relative PDCD4 mRNA expression was measured by using qPCR between control subjects (n=15) and CRSwNP (n=43). **(F)** Correlations between miR-21 and PDCD4 in CRSwNP, R values indicate Spearman correlation coefficients. Mann–Whitney *U* test was used for comparisons between control subjects and CRSwNP. The asterisk indicates statistical significance, *P < 0.05; **P < 0.01; ***P < 0.001.

A previous study confirmed that elevated miR-21 could promote IL-10 production and block NF-κB activation *via* decreasing PDCD4 expression to limit inflammation (28). However, the expression of PDCD4 in nasal mucosa is unclear. Thus, we measured its expression in nasal tissues from control subjects and patients with CRSwNP. IHC staining showed that PDCD4 was expressed in the epithelium and subepithelial layers of both NPs and normal tissues (**Figure 4A**). The MOD analysis of IHC and densitometric analysis of WB demonstrated that the PDCD4 protein remarkably decreased in CRSwNP compared with control subjects (P<0.05) (**Figures 4B-D**). Consistently, PDCD4 mRNA expression was significantly lower in CRSwNP than in control subjects (P<0.001) (**Figure 4E**). Furthermore, we found that PDCD4 expression was negatively correlated with miR-21 in CRSwNP (P<0.01) (**Figure 4F**). The negative correlation between miR-21 and PDCD4 in the nasal epithelium of CRSwNP indicated that miR-21 could regulate PDCD4 expression in nasal epithelial cells.

### MiR-21 Inhibits the Expression of PDCD4 by Targeting Its mRNA

**Figure 5:**
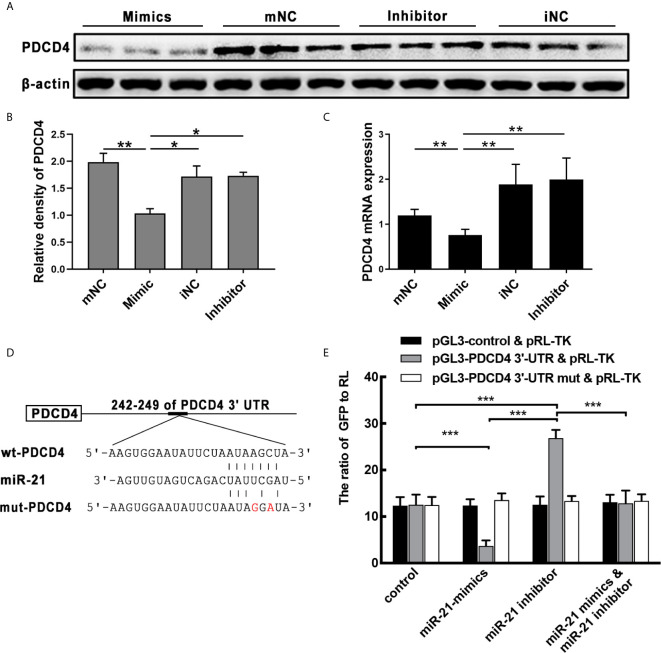
Effect of miR-21 on the expression of PDCD4 *in vitro*. **(A)** HNEpC was transfected with miR-21 mimics (with mNC as control) and inhibitor (with iNC as control) for 24h. PDCD4 protein expression was determined by WB, normalized to β-actin. **(B)** Relative PDCD4 protein expression was quantified by densitometry based on immunoblot images. **(C)** PDCD4 mRNA levels were measured by qPCR. **(D)** The predicted miR-21 binding sites within the 3′UTR of PDCD4 mRNA. **(E)** Double luciferase activity assay of HEK 293 cells. After being co-transfected with the analogue of miR-21: control/miR-21 mimic/miR-21 inhibitor, and the following plasmids: pGL3-3′-UTR of control/WT/mutated PDCD4 vector and the pRL-TK vector, the ratio of GFP to RL was determined. Data were obtained in three independent experiments. One-way ANOVA was used to analyze the difference between multiple groups. The asterisk indicates statistical significance, *P < 0.05; **P < 0.01; ***P < 0.001.

After *in vitro* transfecting, miR-21 mimics or inhibits HNEpC. WB and qPCR analysis showed that PDCD4 protein and mRNA were significantly inhibited by the overexpression of miR-21 (P<0.05) (**Figures 5A-C**). To investigate whether PDCD4 is a target gene of miR-21, we used bioinformatic tools including TargetScan, miRanda, and PicTar to identify the potential miR-21 binding sites in 3′-UTR of PDCD4 mRNA (**Figure 5D**). These results imply that miR-21 could inhibit the expression of PDCD4 mRNA and protein *via* binding its mRNA.

Co-transfection of the PDCD4 luciferase reporter vector and the pRL-TK vector with miR-21 mimic in HEK 293 cells resulted in a significant reduction in luciferase activity relative to the scrambled control, while co-transfection with the miR-21 inhibitor resulted in significantly increased luciferase activity (**Figure 5E**). Furthermore, co-transfection with miR-21 mimic and the miR-21 inhibitor also restored the expression of eGFP (**Figure 5E**). Moreover, luciferase activity was unaffected when the MRE in PDCD4 3′-UTR was mutated (**Figure 5D**), demonstrating that PDCD4 is a direct target of miR-21 (**Figure 5E**). These results confirm PDCD4 as a target of miR-21 *in vitro*. Combining the above results from qPCR and WB of CRSwNP demonstrates that miR-21 plays important roles in the formation of NP through regulating the cytokines expression1 by targeting the PDCD4.

### MiR-21 Suppresses NF-κB P65 Activation and Increases IL-10 Expression in HNEpC

**Figure 6:**
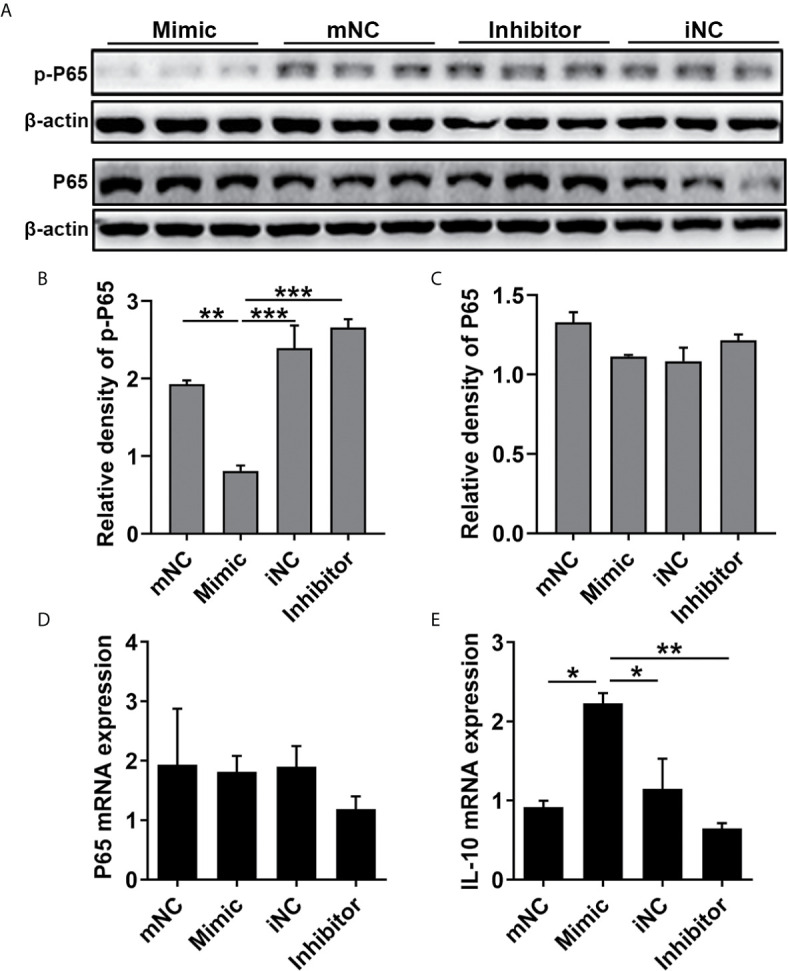
Effect of miR-21 on the expression of NF-κB P65 and IL-10 in HNEpC. HNEpC was transfected with miR-21 mimics (with mNC as control) and inhibitor (with iNC as control) for 24h. **(A)** p-P65 and P65 protein expression was determined by WB, normalized to β-actin. **(B, C)** Relative p-P65 and P65 protein expression were quantified by densitometry based on immunoblot images. **(D, E)** NF-κB and IL-10 mRNA expression were measured by qPCR. Data were obtained in three independent experiments. One-way ANOVA was used to analyze the difference between multiple groups. The asterisk indicates statistical significance, *P < 0.05; **P < 0.01; ***P < 0.001.

To determine the effect of miR-21 on the epithelial cytokines, the protein expression of NF-κB subunit (P65), and the gene expression of P65, IL-1β, IL-6, IL-8, IL-10, IL-25, IL-33, and TSLP were detected. The WB results showed that p-P65 protein expression was significantly inhibited by miR-21 (P<0.05) while P65 protein expression was not affected (P>0.05) (**Figures 6A-C**). The qPCR results showed that the mRNA expression of P65 remained consistent after miR-21 overexpression compared to miR-21 knock-down (**Figure 6D**). Furthermore, the qPCR results revealed that the mRNA expression of IL-10, an anti-inflammatory cytokine, were elevated in the miR-21 mimic group compared with the negative control group (P<0.05), while the miR-21 inhibitor did not affect IL-10 (P>0.05) (**Figure 6E**). No change of other cytokines was measured after miR-21 transfection **(Data not shown)**. Previous studies confirmed that PDCD4 could enhance the phosphorylation of P65 and inhibit the expression of IL-10 (28–31). These results suggest that miR-21 suppresses NF-κB activation and induces IL-10 mRNA expression through decreasing PDCD4 in HNEpC.

### Effect of miR-21 on Cytokines of Nasal Epithelial Cells After Stimulated With SEB and LPS

We next estimated whether miR-21 could regulate the expression of cytokines in HNEpC after SEB or LPS treatment. To evaluate the appropriate dose and time response of SEB and LPS, PDCD4 and p-P65 protein expression were measured at different concentrations and time-points after stimulation (**Supplementary Figures 5, 6**). The results showed 24 h of treatment with 250ng/ml of SEB and 1ug/ml of LPS both could decrease PDCD4 protein expression and induce P65 activation (**Supplementary Figures 5**), **6** . Accordingly, we chose the concentration and time-point of SEB and LPS to stimulate HNEpC.

**Figure 7:**
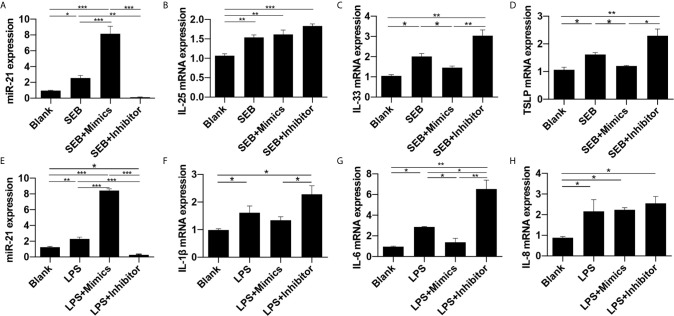
Effect of miR-21 on cytokines in HNEpC after stimulated with SEB and LPS. Expression of **(A)** miR-21, **(B)** IL-25, **(C)** IL-33, and **(D)** TSLP were measured by qPCR in HNEpC after miR-21 transfection followed by SEB treatment. Expression of **(E)** miR-21, **(F)** IL-1β, **(G)** IL-6, and **(H)** IL-8 were measured by qPCR in HNEpC after miR-21 transfection followed by LPS treatment. Data were obtained in three independent experiments. One-way ANOVA was used to analyze the difference between multiple groups. The asterisk indicates statistical significance, *P < 0.05; **P < 0.01; ***P < 0.001.

Then, miR-21 transfection followed by SEB and LPS treatment was conducted. The qPCR results revealed that miR-21 was elevated by SEB and LPS stimulation, and could be enhanced by miR-21 mimics and reduced by miR-21 inhibitor (P<0.05) (**Figures 7A, E**). SEB could increase IL-25, IL-33, and TSLP mRNA expression, and LPS could increase IL-1β, IL-6, and IL-8 mRNA expression in HNEPC (P<0.05) (**Figures 7B-D, F-H**). The effect of SEB on IL-33 and TSLP could be enhanced by miR-21 inhibitor and reduced by miR-21 mimics (P<0.05) (**Figures 7C, D**). Meanwhile, the effect of LPS on IL-1β and IL-6 could be enhanced by miR-21 inhibitor and reduced by miR-21 mimics as well (P<0.05) (**Figures 7F, G**). No alteration of IL-25 or IL-8 resulting from miR-21 was observed (P>0.05) (**Figures 7B, H**). These results indicate that miR-21 plays an anti-inflammation role in HNEpC.

### Effect of miR-21 on Cytokines Of Cultured Nasal Polyp Explants After Stimulated With SEB and LPS

**Figure 8:**
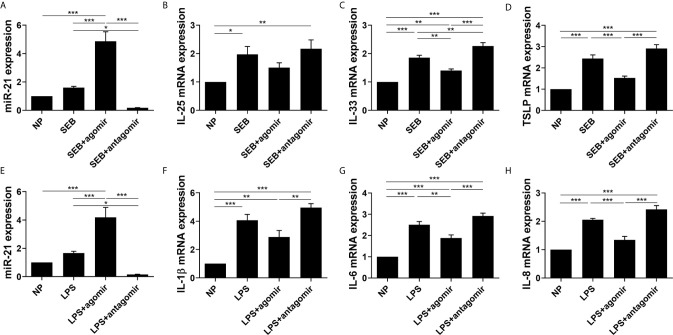
Effect of miR-21 on cytokines in cultured nasal polyp explants after SEB or LPS treatment. Expression of **(A)** miR-21, **(B)** IL-25, **(C)** IL-33, and **(D)** TSLP were measured by qPCR in tissues after miR-21 transfection followed by SEB treatment. Expression of **(E)** miR-21, **(F)** IL-1β, **(G)** IL-6, and **(H)** IL-8 were measured by qPCR in tissues after miR-21 transfection followed by LPS treatment. One-way ANOVA was used to analyze the difference between multiple groups. The asterisk indicates statistical significance, *P < 0.05; **P < 0.01; ***P < 0.001.

We then explored the function of miR-21 on the expression of the cytokines of nasal polyp *via* an *ex vivo* model. MiR-21 expression was confirmed by qPCR (**Figures 8A, E**). SEB could increase IL-25, IL-33, and TSLP mRNA expression, and LPS could increase IL-1β, IL-6, and IL-8 mRNA expression (P<0.05) (**Figures 8B-D, F-H**). The effect of SEB on IL-33 and TSLP could be enhanced by miR-21 antagomir and reduced by miR-21 agomir (P<0.05) (**Figures 8C, D**). Meanwhile, the effect of LPS on IL-6 and IL-8 could be reduced by miR-21 agomir as well (P<0.05) (**Figures 8G, H**). We observed no significant alteration of IL-25 and IL-1β resulting from miR-21 (P>0.05) (**Figures 8B, F**). These results indicate that miR-21 is an anti-inflammation molecular involved in the development of NPs.

### MiR-21 Expression Correlates With Lund-Mackay Scores and Lund-Kennedy Scores

**Figure 9:**
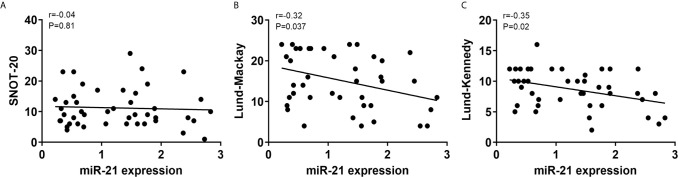
Correlations between miR-21 expression and clinical scores. Correlations between miR-21 expression and SNOT-20 **(A)**, Lund-Mackay **(B)**, and Lund-Kennedy scores **(C)** were investigated in CRSwNP. R-values indicate Spearman correlation coefficients.

To determine whether miR-21 correlated with disease severity in CRSwNP, we assessed the SNOT-20, Lund-Mackay, and Lund-Kennedy scores of CRSwNP. There was no correlation between miR-21 expression and SNOT-20 scores (P>0.05) (**Figure 9A**), whereas miR-21 expression was negatively correlated with Lund-Mackay scores and Lund-Kennedy scores (P<0.05) (**Figures 9B, C**). These results suggested that miR-21 is a brake of inflammation in the development of NPs.

## Discussion

**Figure 10:**
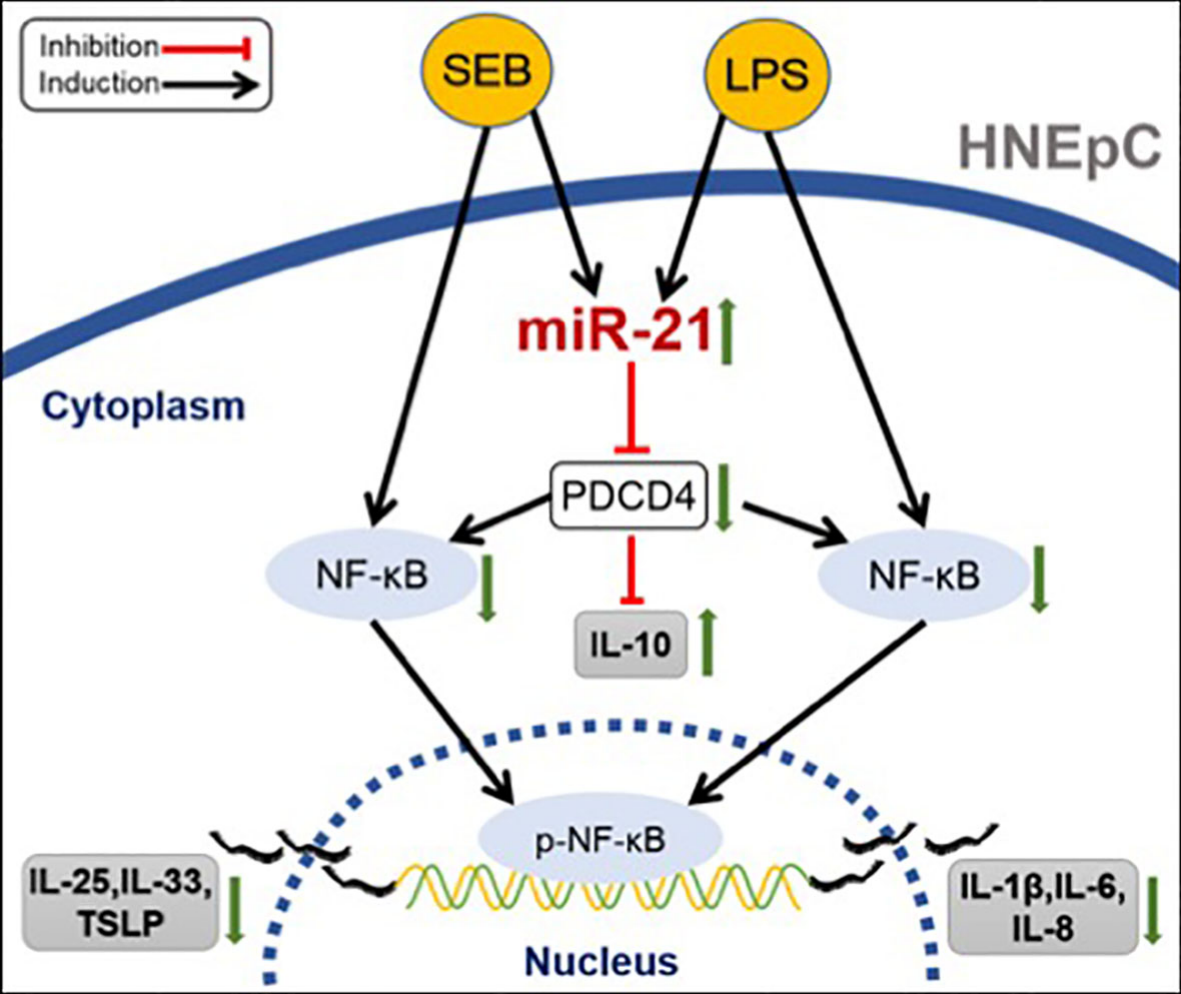
Schematic summary of the anti-inflammatory role of miR-21. In nasal epithelial cells, SEB could promote the expression of IL-25, IL-33, and TSLP, while LPS could enhance the expression of IL-1β, IL-6, and IL-8, both by activating NF-κB. However, both SEB and LPS could upregulate miR-21 expression. Elevated miR-21 would decrease PDCD4 expression to suppress the expression of cytokines in HNEpC by inducing the IL-10 production and inhibiting the activation of NF-κB.

CRSwNP is a heterogeneous disease characterized by distinct expression of inflammatory cytokines (4, 5). Currently, researchers have confirmed that nasal epithelium plays an important role in the formation of NP (3). As the first line of defense of airways, the nasal epithelium is critical for maintaining homeostasis of the underlying tissue mucosa (5). Defects in the function of epithelium cause the accumulation of pathogens in the epithelial layers, and chronic exposure to pathogens induces cytokines secreted by epithelial cells which contribute to inflammation (32–35). Thus, the important treatment strategies for CRSwNP were to control inflammation and restore the function of the nasal mucosal barrier. Our study found that the expression of miR-21 was elevated in CRSwNP compared with control subjects, especially in the epithelial cells of NP. Upregulated miR-21 can inhibit activation of NF-κB P65 and increase IL-10 expression through targeting to PDCD4 in nasal epithelial cells, which could further limit the expression of pro-inflammatory cytokines, as indicated in **Figure 10**. The findings of this study suggest that miR-21 is associated with anti-inflammation and is a prominent negative factor in the inflammation process that attenuates the expression of pro-inflammatory cytokines in nasal polyps.

MiR-21 has been implicated in a broad range of diseases, especially in human malignancies, and a growing number of studies have confirmed that miR-21 is a key switch in inflammation (21). Overexpression of miR-21 was associated with asthma (22), lumbar spinal canal stenosis (36), and dominant negative TGF-β receptor II mice (37). In these conditions, miR-21 promoted inflammation. Conversely, miR-21 could play an anti-inflammation role in lung inflammation (38), wound inflammation (29), and bacterial infection mice (39). Our study confirmed that miR-21 increased in the nasal epithelium of CRSwNP compared with control subjects and that the expression of miR-21 was positively correlated with IL-10, and negatively correlated with IL-6, IL-8, IL-33, and TSLP in the epithelial cells of CRSwNP. It implied that miR-21 might have a function of anti-inflammation in CRSwNP.

Previous studies have confirmed that bolstering miR-21 levels could promote IL-10 production and inhibit the activation of NF-κB *via* decreasing PDCD4 expression (28, 29). We hypothesized that the miR-21/PDCD4/NF-*κ*B and IL-10 pathways also existed in CRSwNP. We found that the protein and mRNA expression of PDCD4 was decreased in CRSwNP compared with control subjects, and PDCD4 mRNA expression was negatively correlated with miR-21. Accordingly, a link between miR-21 and PDCD4 in the epithelium of CRSwNP was identified. Through transfection assay, we found that overexpressed miR-21 could attenuate both the mRNA and protein expression of PDCD4, as indicated in bioinformatic analysis and luciferase activity assay. All these results confirmed that PDCD4 was a target of miR-21 as shown in a previous study by Cohen et al. (39). Furthermore, we explored whether the miR-21/PDCD4 axis regulated NF-κB activation and IL-10 expression in nasal epithelial cells. Through transfection assay, we found that overexpressed miR-21 could suppress P65 activation and increase IL-10 mRNA level, which is in line with previous studies (28–31).

NF-κB is a heterodimer consisting of members of the P50 and P65 families, but the constitutional fraction of P65 acts as the regulator of inflammatory cytokines and induces the inflammatory process (40), and the production of cytokines from nasal epithelial cells was associated with the activation of NF-κB (40–42). Conversely, has also been reported that miR-21 could induce activation of P65 *via* inhibiting PDCD4 expression and could further promote the expression of inflammatory factors (43, 44). Furthermore, a deficiency of PDCD4 could elevate inflammatory cytokine expression as well (45, 46). Therefore, Frederick et al. suggested that variations in the role of miR-21 may be related to the different target mRNAs engaged in different cells (21). Collectively, PDCD4 is a target gene of miR-21 in nasal epithelial cells, and miR-21 could inhibit NF-κB activation and induce IL-10 expression *via* attenuating PDCD4 expression levels.

It was reported that SEB and LPS were ubiquitous toxins of pathogens in the nasal mucosa and were known to participate in the pathogenesis of NP (47, 48). *In vitro*, SEB and LPS can stimulate the release of diverse inflammatory cytokines (49–51). In this study, we chose these two agents to promote cytokine expression in the HNEpC line and *ex vivo* nasal polyp explants. We identified that SEB and LPS could increase the expression of miR-21, and elevate IL-25, IL-33, TSLP, and IL-1β, IL-6, IL-8 expression respectively. Meanwhile, the activation of NF-κB was induced and PDCD4 protein was decreased in HNEpC after treatment with SEB or LPS in HNEpC. Sheedy et al. demonstrated that LPS could decrease PDCD4 protein *via* miR-21 induction (28), similar to our results.

Through increasing or inhibiting miR-21 expression *in vitro* and *ex vivo* treated with SEB or LPS, we found that the expression of pro-inflammatory cytokines could be reduced by up-regulated miR-21 yet promoted by down-regulated miR-21. Considering the pivotal role of NF-κB in cytokine production, we deem that miR-21 inhibits cytokines expression *via* the miR-21/PDCD4/NF-κB pathway. These results indicate that miR-21 could suppress pro-inflammatory cytokine production, thereby inhibiting the development of inflammation in CRSwNP. We also observed a negative relationship between miR-21 expression and the Lund-Mackay scores or the Lund-Kennedy scores, which revealed that miR-21 may play an anti-inflammation role and be associated with the severity of CRSwNP.

In summary, we found that miR-21 could be a protective factor that inhibits the progression of inflammation. Elevated miR-21 could be a brake of inflammation involved in the development of NPs, which inferred that it might be a target in the treatment of CRSwNP.

## Data Availability Statement

All datasets generated for this study are included in the article/**Supplementary Material**.

## Ethics Statement

This study was approved by the Medical Ethics Committee of West China Hospital of Sichuan University (No.: WCH2015-199) and was conducted to the approved institutional guidelines. The patients/participants provided their written informed consent to participate in this study.

## Author Contributions

All authors listed have made a substantial, direct, and intellectual contribution to the work and approved it for publication. RL and JD contributed to the collection of data, data analysis, performed the experiments and manuscript writing. JD also contributed to the idea, experimental design, manuscript submission and revising. JZho, BZ and JZha performed the literature review and assisted in experiments. LB and YL contributed to the provision of study material or patient samples. SL supervised and fund the study and contributed to the final approval of the manuscript. All authors contributed to the article and approved the submitted version.

## Funding

This study was supported by the National Natural Science Fund of China (81970858, 81570900 and 81271058), which supplies the most of funds for the study. Moreover, this study was also supported by the National Natural Science Fund of China (81860186).

## Conflict of Interest

The authors declare that the research was conducted in the absence of any commercial or financial relationships that could be construed as a potential conflict of interest.

## Abbreviations

CRS, chronic rhinosinusitis; NP, nasal polyp; CRSw/sNP, chronic rhinosinusitis with/without nasal polyp; miR, miRNA, microRNA; CT, computed tomography; qPCR, real-time quantitative PCR; ISH, *in situ* hybridization; LNA, locked nucleic acid; IHC, immunohistochemistry; WB, western blotting; MOD, mean optical density; NEC, nasal epithelial cell; HNEpC, Human nasal epithelial cell; IL, interleukin; LPS, lipopolysaccharide; SEB, staphylococcal enterotoxin B.
