# Supplementary material for: Elevated microRNA-21 Is a Brake of Inflammation Involved in the Development of Nasal Polyps
Source: Front Immunol. 2021 Apr 15;12:530488. doi: 10.3389/fimmu.2021.530488 (PMC8082185; doi:10.3389/fimmu.2021.530488)
Supplement: Supplementary file 1 [file DataSheet_1.docx]

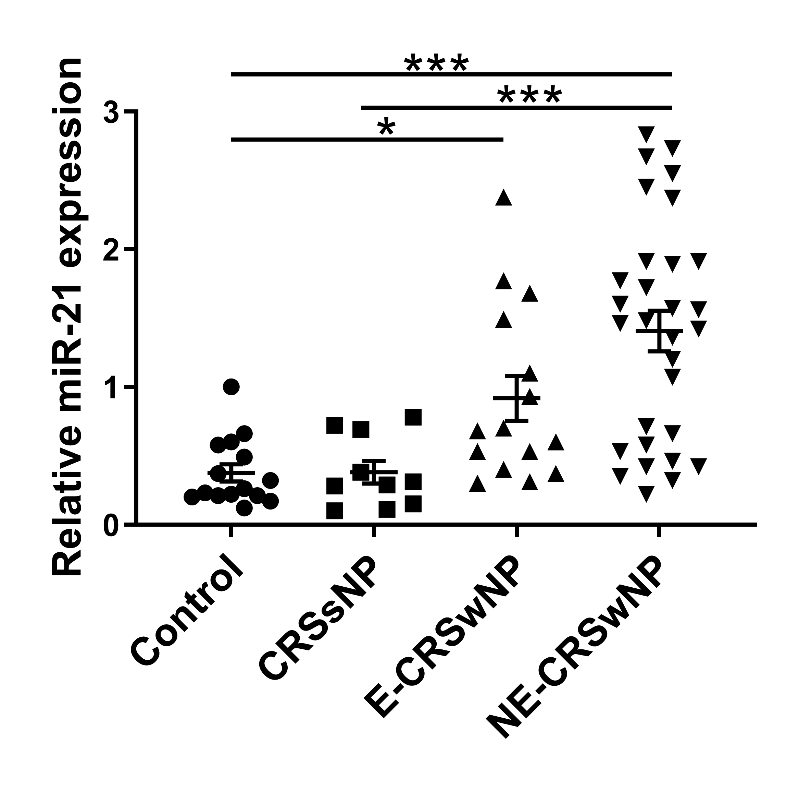


**Supplementary Figure 1.** The expression of miR-21 was measured by qPCR and compared among control subjects, CRSsNP, E-CRSwNP and NE-CRSwNP. Kruskal–Wallis test was used for comparison among multiple groups. Asterisk indicates statistical significance, *P<0.05, ***P<0.001.


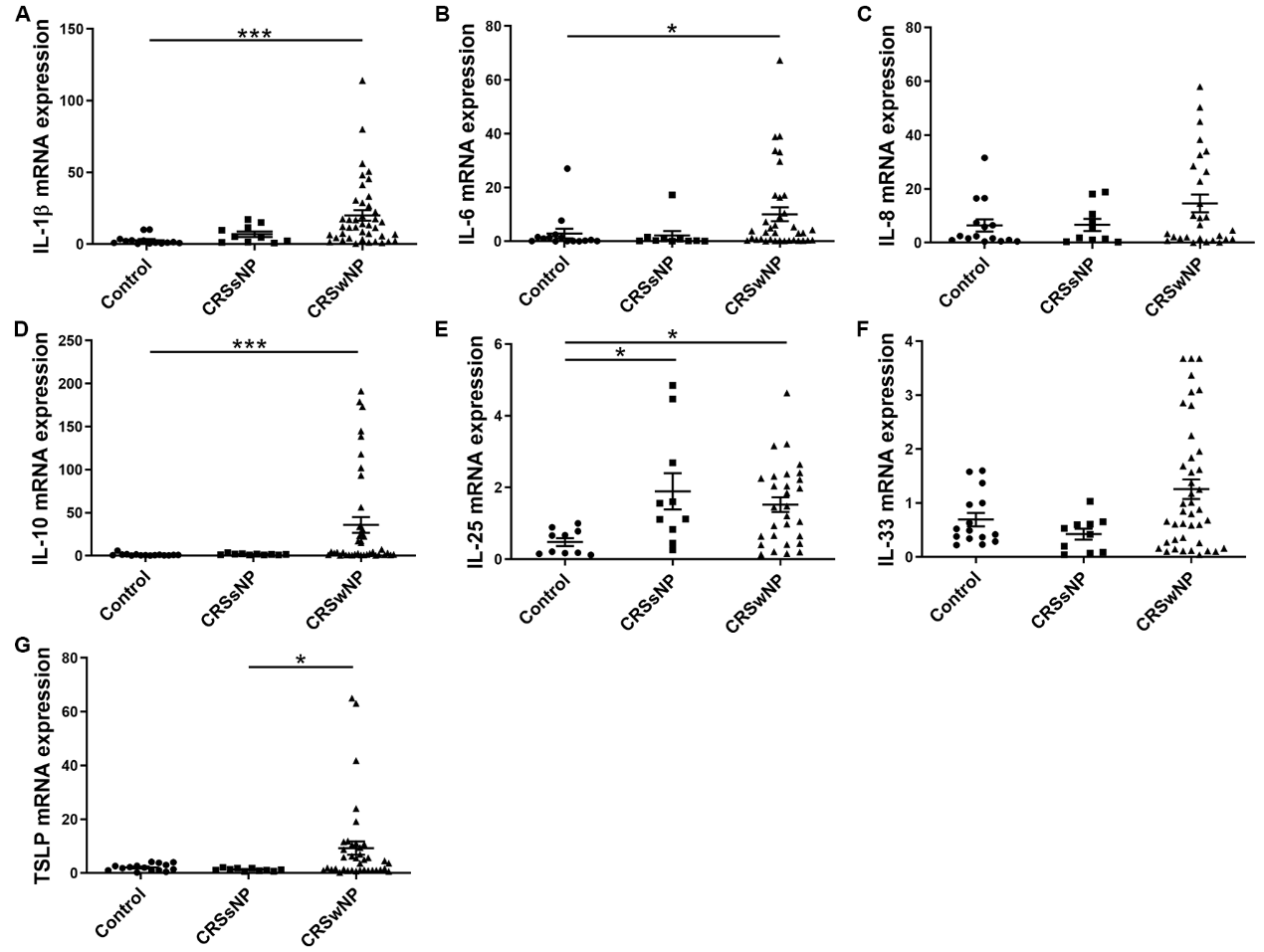


**Supplementary Figure 2.** Relative expression level of epithelial cytokines in nasal mucosa. The mRNA expression of (A)IL-1β, (B)IL-6, (C)IL-8, (D)IL-10, (E)IL-25, (F)IL-33 and (G)TSLP were measured by qPCR and compared among control subjects (n=15), CRSsNP (n=10) and CRSwNP (n=45). Kruskal–Wallis test was used for comparison among multiple groups. Asterisk indicates statistical significance, *P<0.05, ***P<0.001.


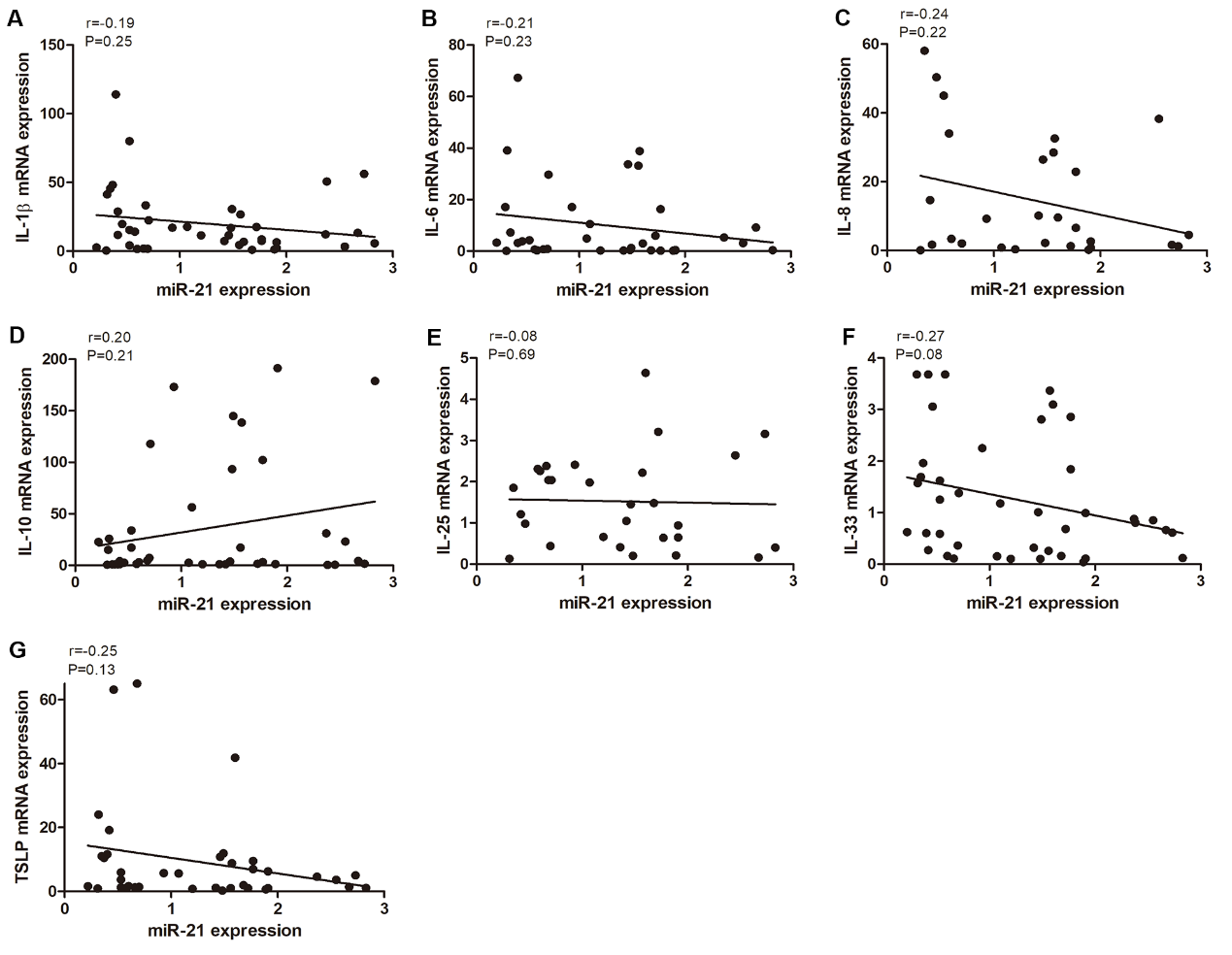


**Supplementary Figure 3.** Correlations between miR-21 expression and cytokines in NPs. Correlations between miR-21 expression and mRNA levels of (A)IL-1β, (B)IL-6, (C)IL-8, (D)IL-10, (E)IL-25, (F)IL-33 and (G)TSLP were investigated in NPs. R values indicate Spearman correlation coefficients.


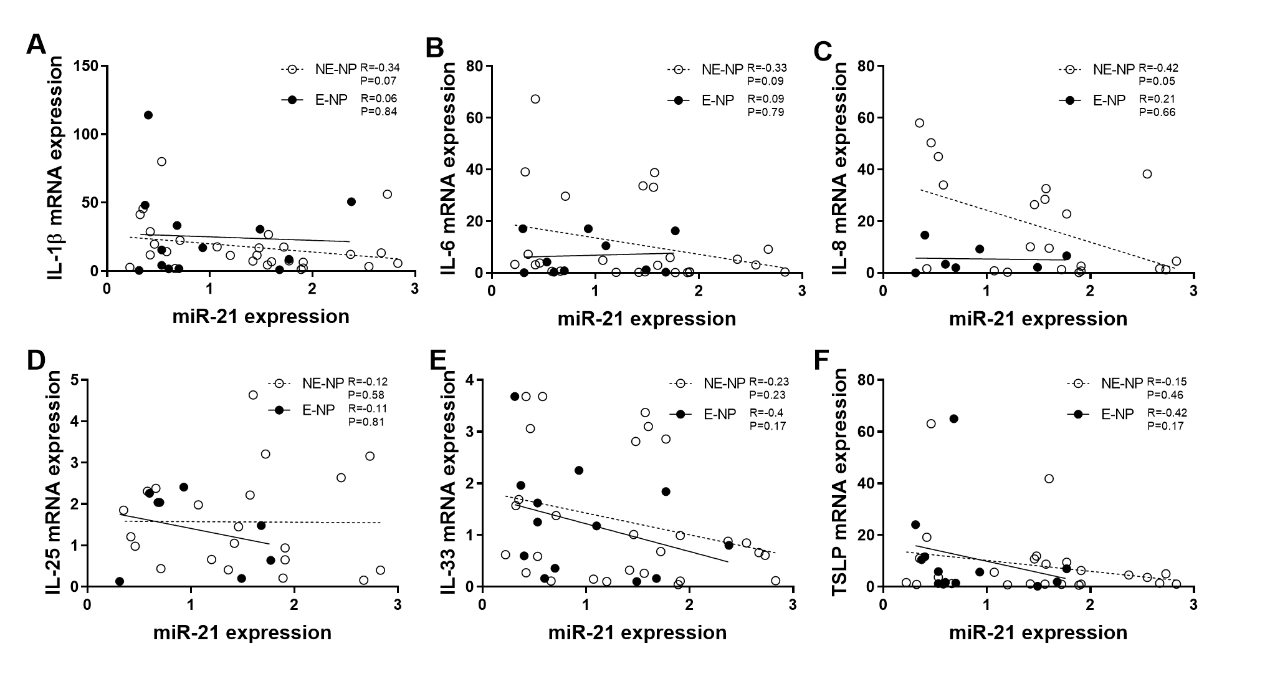


**Supplementary Figure 4.** Correlations between miR-21 expression and cytokines in E-CRSwNP and NE-CRSwNP. Correlations between miR-21 expression and mRNA levels of (A)IL-1β, (B)IL-6, (C)IL-8, (D)IL-25, (E)IL-33 and (F)TSLP were investigated. R values indicate Spearman correlation coefficients.


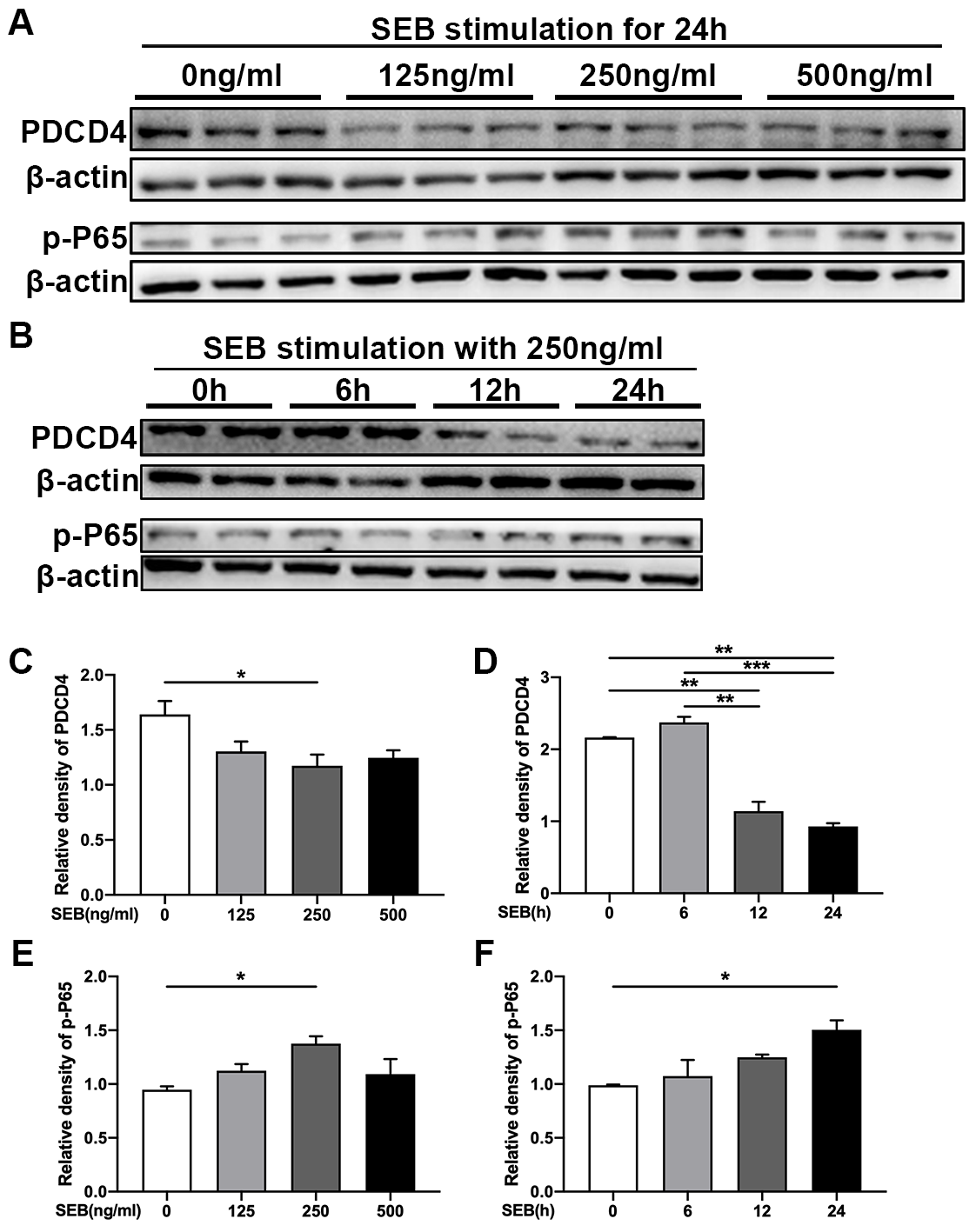


**Supplementary Figure 5.** Effect of SEB on the expression of PDCD4 and the activation of P65 in HNEpC. (A) PDCD4 and p-P65 protein expression was determined by WB in HNEpC treated with SEB (0/125/250/500 ng/ml) for 24h. (B) PDCD4 and p-P65 protein expression was determined by WB in HNEpC treated with SEB (250 ng/ml) for 0-24h. (C, D) Relative PDCD4 protein expression was quantified by densitometry based on immunoblot image A and B. (E, F) Relative p-P65 protein expression was quantified by densitometry based on immunoblot image A and B. Data were obtained in three (concentration curve) or two (time course) independent experiments. One-way ANOVA was used to analyze the difference between multiple groups. Asterisk indicates statistical significance, *P<0.05; **P<0.01; ***P<0.001.


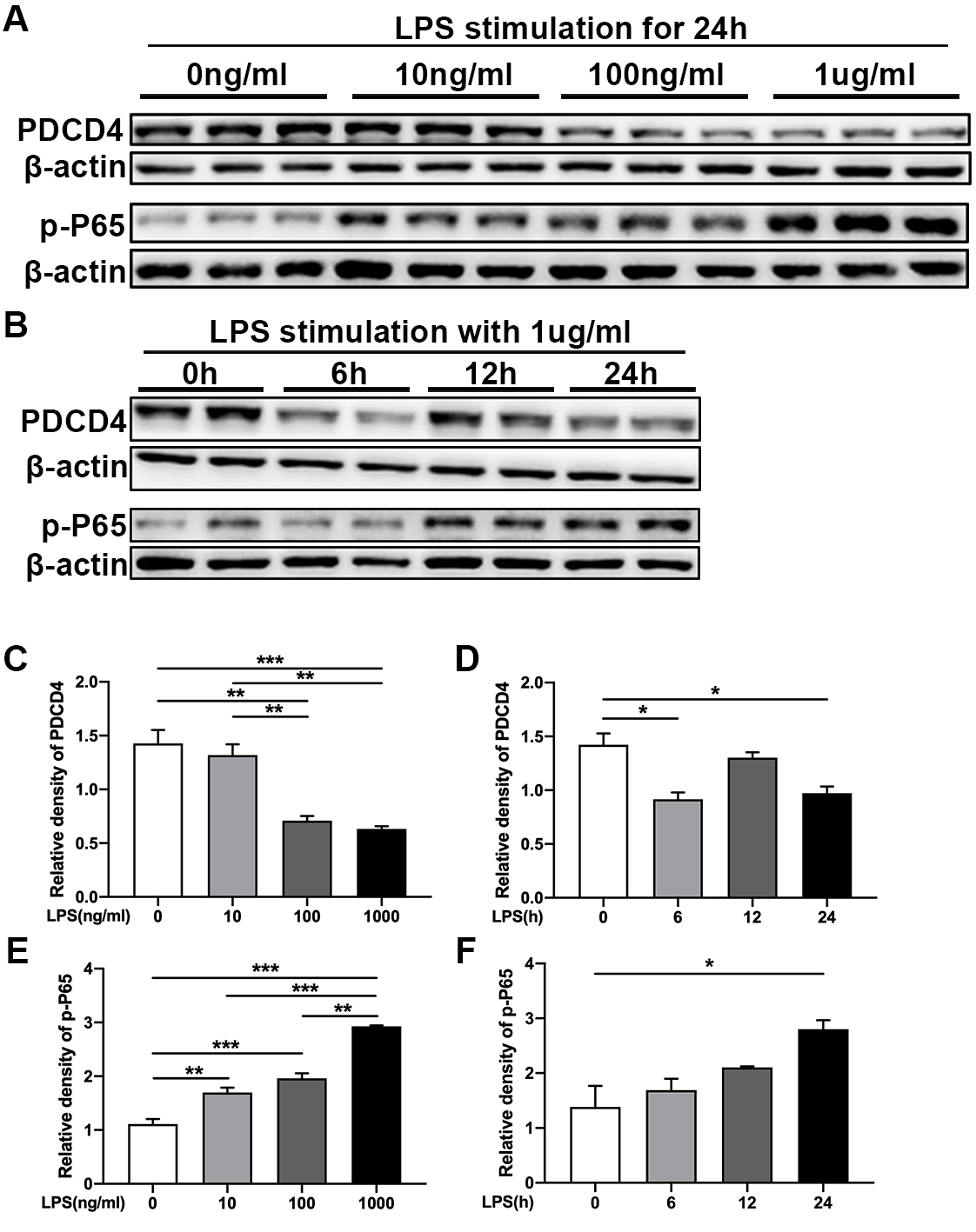


**Supplementary Figure 6.** Effect of LPS on the expression of PDCD4 and the activation of P65 in HNEpC. (A) PDCD4 and p-P65 protein expression was determined by WB in HNEpC treated with LPS (0/10/100/1000 ng/ml) for 24h. (B) PDCD4 and p-P65 protein expression was determined by WB in HNEpC treated with LPS (1 ug/ml) for 0-24h. (C, D) Relative PDCD4 protein expression was quantified by densitometry based on immunoblot image A and B. (E, F) Relative p-P65 protein expression was quantified by densitometry based on immunoblot image A and B. Data were obtained in three (concentration curve) or two (time course) independent experiments. One-way ANOVA was used to analyze the difference between multiple groups. Asterisk indicates statistical significance, *P<0.05; **P<0.01; ***P<0.001.
